# Distal femoral replacement with the MML system: a single center experience with an average follow-up of 86 months

**DOI:** 10.1186/s12891-017-1570-9

**Published:** 2017-05-22

**Authors:** Andreas Toepfer, Norbert Harrasser, Paul-Ruben Schwarz, Florian Pohlig, Ulrich Lenze, Heinrich M. L. Mühlhofer, Ludger Gerdesmeyer, Ruediger von Eisenhart-Rothe, Christian Suren

**Affiliations:** 1 Department of Orthopedics and Sports Orthopedics, Technical University of Munich, 81547 Munich, Germany; 2 KH Garmisch-Partenkirchen, Garmisch-Partenkirchen, Germany; 3 Department of Orthopaedic Surgery and Traumatology, University of Schleswig Holstein, Lübeck, Germany

**Keywords:** Distal femoral replacement, Revision arthroplasty, Infection, Megaprosthesis, Sarcoma

## Abstract

**Background:**

The aim of this study was to compare the functional outcomes and complication rates after distal femoral replacement (DFR) performed with the modular Munich-Luebeck (MML) modular prosthesis (ESKA/Orthodynamics, Luebeck, Germany) in patients being treated for malignant disease or failed total knee arthroplasty.

**Methods:**

A retrospective review of patient charts and a functional investigation (involving Musculoskeletal Tumor Society Score [MSTS], American Knee Society Score [AKSS], Oxford Knee Score [OKS], Western Ontario and McMaster Universities Osteoarthritis Index [WOMAC], Toronto Extremity Salvage Score [TESS], the 12-Item Short-Form [SF-12] Health Survey, and a failure classification system developed by Henderson et al.) of DFR cases from 2002 to 2015 were conducted. The indications for DFR were malignant tumor resection in the femur (*n* = 20, group A) or failure of revision total knee arthroplasty without a history of malignant disease (*n* = 16, group B).

**Results:**

One-hundred and twenty-nine patients were treated during the study period. Of these, 82 were analyzed for complications and implant-survival. Further, 36 patients were available for functional assessment after a mean follow-up of 86 months (range: 24–154). There were 75 complications in total. The overall failure rate for DFR was 64.6% (53/82 patients). The most common failure mechanisms were type III (mechanical failure), followed by type I (soft tissue) and type II (aseptic loosening). The mean MSTS score (out of 30) was 17 for group A and 12 for group B. All the clinical outcome scores revealed an age-dependent deterioration of function.

**Conclusion:**

DFR is an established procedure to restore distal femoral integrity. However, complication rates are high. Post-procedure functionality depends mainly on the patient’s age at initial reconstruction.

## Background

With improved survival for primary bone malignancies resulting from modern chemotherapy regimens, the development of limb-salvage procedures has flourished. In the lower extremity, where the primary function of the skeleton is to support body weight and allow ambulation, reconstruction of bone defects is of major interest to preserve its function. Many surgical options for the reconstruction and stabilization of massive bone defects around the knee joint have been described and include biologic options with autografts and allografts or the use of tumor endoprostheses [1]. The evolution of distal femoral replacements (DFR) has gone from custom-made devices to modern modular implants and more recently, to the addition of antibacterial coatings [2].

Apart from bone defects caused by tumors, failed revision arthroplasty has become another challenging indication for the use of DFR. Although results of current tumor megaprostheses have been discussed consistently in recent literature [3–14], various aspects affecting the outcome have not been uncovered yet: It is still unclear which patients are at risk to experience low functional outcome after this procedure. Hence, it is not well understood whether complications after DFR depend on the indication for surgery or, for instance, the patients’ age. Further, there have been but few reports that evaluated functional outcomes after these reconstructions with the modular Munich-Luebeck (MML) knee prosthesis for various indications [15, 16].

Therefore, the aim of the present study was to report the outcomes and complications in patients treated with MML DFR from one orthopedic center. We asked the following questions: (1) What are the functional outcomes of modular knee replacements with DFR? (2) Do complications vary with the indication for this procedure (malignant disease vs. revision arthroplasty)?

## Methods

**Fig. 1:**
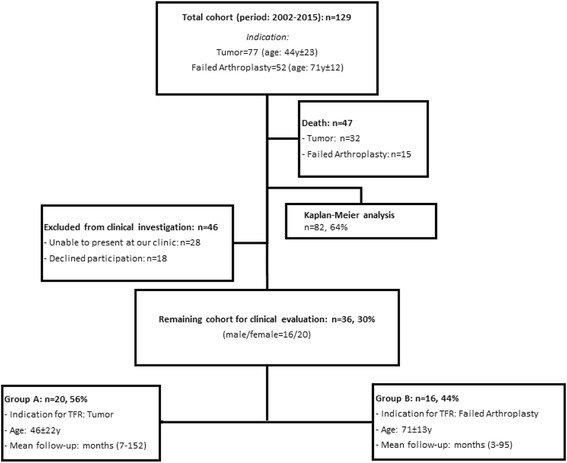
Total cohort and patients included in the study groups

Approval of the institutional review board and written consent from each subject prior to inclusion were obtained before initiating the study. We retrospectively reviewed our institution’s database for patients who underwent reconstruction of the distal femur and/or proximal tibia due to tumors or failed revision arthroplasties from January 2002 to January 2015. Reconstruction of the defect was carried out with the modular knee prosthesis MML (ESKA/Orthodynamics, Luebeck, Germany) comprising a fully constrained total knee system with the possibility to augment distal femoral defects. We identified 129 patients (male/female = 55/74) with 129 DFRs. Forty-seven of the 129 patients had died at the time of our chart review, and another 46 were excluded from clinical investigation because they were not able to present at our clinic or declined to participate in the clinical follow-up part of this study. Nevertheless, they gave consent to be included in the prosthesis survival analysis. Thus, 36 patients were included in our study for clinical investigation and 82 patients for the survival analysis (Fig. 1). Demographic data of the cohort are given in Table 1. Patients were subdivided into groups A and B according to the indication for DFR: malignant musculoskeletal disease (group A; *n* = 20, mean age 46,2 ± 22,1 years) or failed revision arthroplasty (group B; *n* = 16, mean age 71,0 ± 13,3 years). Surgical details, follow-up intervals and examinations, complications, and functional scores for massive bone defect reconstruction (Musculoskeletal Tumor Society [MSTS] score) were recorded. Additionally, functional scores evaluating results after knee surgeries (American Knee Society Score [AKSS], Oxford Knee Score [OKS], Western Ontario and McMaster Universities Osteoarthritis Index [WOMAC], Toronto Extremity Salvage Score [TESS]), pain (numeric pain rating scale [NRS]), and overall health-related-quality of life (12-Item Short-Form [SF-12] Health Survey) were analyzed. Complications were classified according to the five modes of failure for megaprostheses established by Henderson et al. (Table 2) [17, 18].

**Table 1:** Categorization of failure modes according to *Henderson* [17]

Category	Type	Subgroup
Mechanical	I. Soft tissue failure	A: instability due to tendon/muscle rupture B: aseptic wound dehiscence
	II. Aseptic loosening	A: <2 years after implantation B:>2 years after implantation
	III. Structural failure	A: prosthetic failure B: periprosthetic fracture
Non-mechanical	IV. Periprosthetic infection	A: <2 years after implantation B:>2 years after implantation
	V. Tumor progression with contamination of prosthesis	A: soft tissue tumor B: bone tumor

**Table 2:** MSTS score with subdomains of both groups

		General criteria			Limb-specific criteria			Score
		Pain	Function	General acceptance	Supports	Walking ability	Gait	
Group A	Preoperative	3,2	3,1	3,1	3,7	4,2	3,8	21 ± 8
	Postoperative	3,5	3,0	2,6	3,0	3,1	2,2	17 ± 8
	*p*-value	0,8	0,86	0,74	0,64	0,51	0,52	0,14
Group B	Preoperative	2,25	2,0	2,6	3,4	3,1	2,2	16 ± 7
	Postoperative	2,9	1,9	1,5	1,8	1,9	1,6	12 ± 9
	*p*-value	0,6	0,83	0,43	0,09	0,08	0,32	0,23

### Statistics

Kaplan–Meier survivorship analysis of 82 prosthetic devices was performed. All data are reported as the mean (with standard deviation) or percentage, where applicable. Comparisons of patient-reported outcomes were performed using a *t*-test for unpaired samples. Where applicable, the Mann–Whitney *U* test for independent samples was used. Statistical significance was set at *p* < 0.05. Correlations between age and clinical outcome was performed with linear regression analysis and six age-matched groups (*n* = 6 per group), and the r^2^ value is reported. Statistical analysis was performed using SPSS 2.0 (IBM, Armonk, NY, USA).

## Results

### Clinical outcome

Overall, patients in our series had a mean numeric pain rating scale of 4.8 preoperatively (group A: 3.6; group B: 6.2; *p* < 0.05) and 3.4 after DFR (group A: 2.3, group B: 4.9; *p* < 0.05). The differences between pre- and postoperative NRS values within each group were not significant (group A: *p* = 0.283; group B: *p* = 0.304).

The mean preoperative MSTS score of group A (20.9 ± 7.9) was significantly higher (*p* < 0.05) than in group B (15.5 ± 6.9) (Table 3). Postoperatively, both groups showed a reduction of MSTS score compared to their preoperative value (group A: 17.2 ± 8.1; group B: 11.6 ± 8.8). The difference of these postoperative values was not significant (*p* = 0.138) between the groups.

**Table 3:** Functional outcome results of both groups

Scores	Group A [Value, SD]	Group B [Value, SD]	*p*-value
WOMAC	26,2 ± 20,8	49,6 ± 26,2	0,011
AKS	74,0 ± 23,0	46,9 ± 32,1	0,012
TESS	80,2 ± 26,5	46,7 ± 33,4	0.008
OKS	31,5 ± 11,1	19,3 ± 12,4	0.008
SF-12			
Physical subdomain	39,6 ± 11,3	31,9 ± 10,0	0.06
Mental subdomain	49,9 ± 9,8	38,8 ± 11,9	0.006

*p* < 0.05 = significant; SD: standard deviation

WOMAC (Western Ontario and McMaster Universities Osteoarthritis Index): 96 (poor)-0 (very good)

AKS (American Knee Society Score): <60: poor; 60 – 69: fair; 70–79: good; 80–100: excellent

TESS (Toronto Extremity Salvage Score): 0 (poor)-100 (very good)

OKS (Oxford knee score): <19: poor; 20–29: fair; 30–39: good; 40–48: very good

SF-12 (Short Form 12 Health Survey): healthy controls > 50

Clinical outcome data computed by AKSS, TESS, OKS, WOMAC, as well as results of SF-12 analysis are provided in Table 3. Correlation of functional outcome values of AKSS, TESS, NRS, and OKS showed high dependence on the age with r^2^ > 0.8 for all four scores: the higher the age, the worse the clinical outcome (Table 4). Physical subdomain of SF-12 also showed an age-dependent correlation, with all values being worse than the general population. On the other hand, the mental subdomain was not significantly reduced in comparison to the general population up to group 3 (mean age, 54.6 years); group 4 to 6 showed significantly reduced values. Analyses of the age-dependent correlation of clinical outcome evaluated with OKS and AKSS showed that patients in group B had a more pronounced deterioration of function than patients in group A; however, this difference was not statistically significant.

**Table 4:** Comparison of age-matched groups and clinical outcome

Group (n. of patients)	Mean age (years)	AKS	OKS	TESS	SF 12 KSS	SF12 PSS	NRS
1 (6)	23,5	90,00	88	0,89	51,41	50,38	0,83
2 (6)	36,6	78,33	60	0,65	37,80	48,29	2,33
3 (6)	54,6	60,00	50	0,58	34,04	49,03	2,67
4 (6)	69,0	58,33	51	0,56	33,35	45,31	3,83
5 (6)	75,7	33,33	37	0,33	28,72	39,13	5,67
6 (6)	83,3	51,43	36	0,40	30,37	36,03	5,86

### Complications

Eighty-two patients were analyzed for complications, of which 53 patients showed 75 complications, and 29 patients had no complications. Further analysis revealed 36 patients with one complication, 13 patients with two, three patients with three, and one patient with four. Comparing the rate of complications between both groups, 32 patients with tumors had 45 complications, and 21 patients with failed arthroplasty had 30 complications (Table 5).

**Table 5:** Number of complications in the present series as classified according to Henderson et al. [17]

Type (Henderson)	Number of complications	Group A (Tumor)	Group B (Failed arthroplasty)
Type I	17 (22.7%)	11 (24.4%)	6 (20%)
A	10 (13.3%)	5 (11.1%)	5 (16.7%)
B	7 (9.3%)	6 (13.3%)	1 (3.3%)
Type II	15 (20%)	8 (17.8%)	7 (23.3%)
A	7 (9.3%)	4 (8.9%)	3 (10%)
B	8 (10.7%)	4 (8.9%)	4 (13.3%)
Type III	28 (37.3%)	18 (40%)	10 (33.3%)
A	17 (22.7%)	13 (28.9%)	4 (13.3%)
B	11 (14.7%)	5 (11.1%)	6 (20%)
Type IV	13 (17.3%)	6 (13.3%)	7 (23.3%)
A	11 (14.7%)	5 (11.1%)	6 (20%)
B	2 (2.7%)	1 (2.2%)	1 (3.3%)
Type V	2 (2.7%)	2 (4.4%)	0 (0%)
A	1 (1.3%)	1 (2.2%)	0 (0%)
B	1 (1.3%)	1 (2.2%)	0 (0%)
Total	75 (100%)	45 (100%)	30 (100%)

% of total complications are put in parentheses

### Implant survival analysis

Eighty-two patients were included in the survivorship analysis. Implant failure was defined as partial or complete exchange of the megaprosthesis or amputation due to implant-related complications. Mean implant survival was 81.8 ± 7.3 months, with 53% of all patients living with the initially implanted device after 60 months. Implants of patients with tumors had a mean survival of 81.2 ± 9.7 months, while those of patients with failed arthroplasty showed a mean survival of 76.5 ± 9.7 months (*p* = 0.958).

## Discussion

To our best knowledge, this is the largest case series of DFR with the MML-System published to date.

Our study has several limitations that must be taken into consideration. First, the sample size in our report is small in terms of statistical analysis, and the analysis is retrospective. Second, there is no comparison between defect reconstruction with DFR and other surgical treatments such as allograft reconstruction or amputation. Third, the procedures were performed by multiple surgeons, so individual surgical techniques might have affected the overall results.

To address our first and second questions, we detected a high overall complication rate of 64%, considerably higher than the complication rate after standard revision arthroplasty of 24.1% reported by Werner et al. in a large cohort of 28,812 patients [1].

The main local complications in our cohort were Henderson type III (structural failure) and type I (soft tissue failure) followed by Henderson type II (aseptic loosening) and type IV (deep infections). Regarding type III complications, problems associated with the prosthesis (type IIIA) were more frequent than periprosthetic fractures (type IIIB).

Starting in the late 1990s, bolt breakage at the level of the hinged knee module was reported in the first-generation design of the MML prosthesis. Hence, the design was changed and the second-generation prostheses had strengthened central axis bolts [16]. In our cohort, only second generation prostheses were implanted. Nevertheless, some of the patients in the tumor group of our cohort had up to three incidences of bolt breakage, none necessitating prosthesis exchange. A possible explanation for tumor patients exhibiting prosthesis failure more frequently may be their lower mean age of 46 years. Younger patients after primary reconstruction are more likely to undertake normal activities including sports than older people with a history of revision arthroplasty.

In our cohort, patients with DFR after tumor resection were more likely to have wound complications than patients with DFR after failed arthroplasty. Due to different surgical principles between the two groups, local wound problems may occur more often after radical tumor surgery, as it is a limb-salvage operation with the primary aim of tumor-free survival. We assume that the extensive resection of a tumor is a predisposing factor for wound complications because of compromised blood circulation in the remaining soft tissue. The most frequent Henderson type IB complication in our cohort was rupture of the patellar tendon. The probability for this complication did not differ between the two groups. This complication is inconsistently discussed in literature: While Bus et al. reported no rupture over 15 years using the Modular Universal Tumor And Revision System (MUTARS), Ruggieri et al. described this problem as one of the main complications of DFR in their cohort [2, 3].

Our results concerning type IV failure rates are comparable to cohorts of other research groups [2, 7–9]. The higher rate among failed arthroplasty cases might be a result of the number of previous surgeries in that cohort leading to impaired tissue coverage.

Henderson type II (aseptic loosening) occurred in 17.8% cases after primary reconstruction and in 23.3% cases after failed revision arthroplasty. This is comparable to most long-term follow-up studies where this type of failure was reported at a rate of 2.4–15.4% for cemented [13, 18–20] and 0–8% for cementless implants [21–24].

We did not find significant differences concerning the likelihood of complications in our groups; this may be caused by the limited number of patients and resulting lack of statistical power. Mean implant survival was comparable in both groups: 81.8 ± 7.3 months (tumor) vs. 76.5 ± 9.7 months (arthroplasty). Data from the literature show heterogeneous 5- and 10-year survival rates, ranging from 25–93% (Table 6).

**Table 6:** Literature overview on distal femoral replacement after tumor surgery

Study	No. of implants	Mean follow-up (years)	Functional Assessment	Implant survival		
				5 years	10 years	15 years
Batta et al. [14]	69	10.4	N/A	73%	65%	55%
Biau et al.^a^ [13]	91	5.2	N/A	76%	45%	29%
Bickels et al. [12]	110	7.8	N/A	93%	88%	N/A
Bus et al.^a^ [25]	110	7.2	N/A	89% at follow-up		
Coathup et al. [10]	61	8.5	N/A	89%	84%	75%
Griffin et al.^a^ [9]	99	6.1	N/A	82%	N/A	N/A
Kinkel et al.^a^ [8]	77	3.8	N/A	57%	N/A	N/A
Morgan et al.^a^ [7]	105	4.8	N/A	73%	59%	N/A
Myers et al.[6]	335	12	N/A	83%	67%	51%
Pala et al.^a^ [5]	247	4	N/A	70% at 4 years	48% at 8 years	
Plötz et al.^a^ [15]	60	4.9	N/A	34%	25%	N/A
Ruggieri et al.^b^ [4]	669	11	N/A	N/A	80%	55% at 20 years
Schwartz et al. [3]	186	8	N/A	N/A	77%	N/A

^a^Study reports on distal femoral and proximal tibial replacement

^b^Study reports on distal femoral, total femoral, and proximal tibial replacement

Our results showed a reduction of pain using the NRS score from 4.8 preoperatively to 3.4 postoperatively. Patients in the failed arthroplasty group had significantly higher preoperative pain levels than the tumor group. This is important because according to Robinson et al., a low preoperative quality of life correlates with lower postoperative function [10].

Functional outcome measurement with respect to the MSTS score, the only established score for evaluation of massive bone reconstructions, revealed significantly higher average preoperative values in the tumor group than in the revision arthroplasty group. Additionally, there was a significant difference of 25 years in the mean ages of the two groups. This is the most influential factor on MSTS score differences between the groups as shown in linear regression analysis. We analyzed all relevant clinical scores for the knee (AKSS, TESS, OKS, WOMAC) as well as the SF-12. Our main finding indicated that the functional outcome after DFR is strongly related to the age of the patient. We compared age-matched groups and clinical outcome in AKSS, OKS, TESS, and NRS. With an r^2^ > 0.8, we demonstrate that high age is related to worse clinical outcomes after DFR. Pala et al. stated that the preoperative quality of life and preoperative function are crucial factors for the outcome in revision hip arthroplasty [5]. We observed similar effects in our revision arthroplasty group. Additionally, the mental and physical condition scaled in the SF-12 is strongly age related, with better results among younger patients. Review of the literature regarding functional outcome measures after DFR revealed data given in Table 7.

**Table 7:** Literature overview on distal femoral replacement after tumor surgery

Study	No. of implants	Mean follow-up (months)	Postoperative Functional Assessment	Implant survival
Back et al. [26]	32	58	KSS: 95	84%
Barrack et al. [27]	16	51	KSS: 131	94%
Barrack et al. [28]	23	58	KSS: 133	96%
Berend et al. [29]	39	46	KSS: 123	87%
Jones et al. [30]	16	47	KSS: 137	96%
Jones et al. [31]	30	49	KSS: 134	88%
Lombardi et al. [32]	113	25	HSS: 73	85%
Petrou et al. [33]	100	132	KSS: 163	87%
Pour et al. [34]	44	50	KSS 117	70%
Pradham et al. [35]	51	48	HSS: 72	N/A
Rand et al. [36]	38	50	N/A	48%
Springer et al. [37]	69	75	KSS : 100	67%
Springer et al. [38]	26	59	KSS: 101	73%
Utting and Newman [39]	30	36	KSS: 106	70%
Westrich et al. [40]	24	33	KSS: 128	92%

*KSS* Knee society clinical score (range: 0–200), *HSS* Hospital for special surgery score (range: 0–100)

## Conclusion

DFR is an established procedure to restore distal femoral integrity. This retrospective analysis confirms the high incidence of implant-related complications and failures in DFR for complex oncological and non-oncological lower limb salvage as already outlined by previous studies with different prosthetic systems. Structural failure, soft tissue problems, and prosthetic joint infections are the complications that surgeons face when implanting DFR, without significant differences in oncologic and non-oncologic patients. Functional and mental outcomes seem to depend mainly on the patients’ age at reconstruction, showing significantly better results in younger oncologic patients receiving reconstruction after tumor surgery. Orthopedic surgeons and patients should be aware of potentially high complication rates and age-related functional outcome of this procedure.

## Abbreviations

AKS: American knee society score
DFR: Distal femoral replacement
OKS: Oxford knee score
SF-12: Short form 12 health survey
TESS: Toronto extremity salvage score
WOMAC: Western Ontario and McMaster Universities Osteoarthritis Index

## References

[1] Lombardi AV, Berend KR (2006). The shattered femur: radical solution options. J Arthroplasty.

[2] Pennekamp PH, Wirtz DC, Durr HR (2012). [Proximal and total femur replacement]. Oper Orthop Traumatol.

[3] Schwartz AJ, Kabo JM, Eilber FC, Eilber FR, Eckardt JJ (2010). Cemented distal femoral endoprostheses for musculoskeletal tumor: improved survival of modular versus custom implants. Clin Orthop Relat Res.

[4] Ruggieri P, Mavrogenis AF, Pala E, Abdel-Mota’al M, Mercuri M (2012). Long term results of fixed-hinge megaprostheses in limb salvage for malignancy. Knee.

[5] Pala E, Trovarelli G, Calabro T, Angelini A, Abati CN, Ruggieri P (2015). Survival of modern knee tumor megaprostheses: failures, functional results, and a comparative statistical analysis. Clin Orthop Relat Res.

[6] Myers GJ, Abudu AT, Carter SR, Tillman RM, Grimer RJ (2007). Endoprosthetic replacement of the distal femur for bone tumours: long-term results. J Bone Joint Surg (Br).

[7] Morgan HD, Cizik AM, Leopold SS, Hawkins DS, Conrad EU (2006). Survival of tumor megaprostheses replacements about the knee. Clin Orthop Relat Res.

[8] Kinkel S, Lehner B, Kleinhans JA, Jakubowitz E, Ewerbeck V, Heisel C (2010). Medium to long-term results after reconstruction of bone defects at the knee with tumor endoprostheses. J Surg Oncol.

[9] Griffin AM, Parsons JA, Davis AM, Bell RS, Wunder JS (2005). Uncemented tumor endoprostheses at the knee: root causes of failure. Clin Orthop Relat Res.

[10] Coathup MJ, Batta V, Pollock RC, Aston WJ, Cannon SR, Skinner JA, Briggs TW, Unwin PS, Blunn GW (2013). Long-term survival of cemented distal femoral endoprostheses with a hydroxyapatite-coated collar: a histological study and a radiographic follow-up. J Bone Joint Surg Am.

[11] Bus MP, van de Sande MA, Fiocco M, Schaap GR, Bramer JA, Dijkstra PD (2017). Erratum to: what Are the long-term results of MUTARS(R) modular endoprostheses for reconstruction of tumor resection of the distal femur and proximal tibia?. Clin Orthop Relat Res.

[12] Bickels J, Wittig JC, Kollender Y, Henshaw RM, Kellar-Graney KL, Meller I, Malawer MM (2002). Distal femur resection with endoprosthetic reconstruction: a long-term followup study. Clin Orthop Relat Res.

[13] Biau D, Faure F, Katsahian S, Jeanrot C, Tomeno B, Anract P (2006). Survival of total knee replacement with a megaprosthesis after bone tumor resection. J Bone Joint Surg Am.

[14] Batta V, Coathup MJ, Parratt MT, Pollock RC, Aston WJ, Cannon SR, Skinner JA, Briggs TW, Blunn GW (2014). Uncemented, custom-made, hydroxyapatite-coated collared distal femoral endoprostheses: up to 18 years’ follow-up. Bone Joint J.

[15] Plotz W, Rechl H, Burgkart R, Messmer C, Schelter R, Hipp E, Gradinger R (2002). Limb salvage with tumor endoprostheses for malignant tumors of the knee. Clin Orthop Relat Res.

[16] Gerdesmeyer L, Topfer A, Kircher J, Grundei H, Diehl P (2006). [The modular MML revision system in knee revision and tumor arthroplasty]. Orthopade.

[17] Henderson ER, O’Connor MI, Ruggieri P, Windhager R, Funovics PT, Gibbons CL, Guo W, Hornicek FJ, Temple HT, Letson GD (2014). Classification of failure of limb salvage after reconstructive surgery for bone tumours : a modified system Including biological and expandable reconstructions. Bone Joint J.

[18] Ahlmann ER, Menendez LR, Kermani C, Gotha H (2006). Survivorship and clinical outcome of modular endoprosthetic reconstruction for neoplastic disease of the lower limb. J Bone Joint Surg (Br).

[19] Guo W, Ji T, Yang R, Tang X, Yang Y (2008). Endoprosthetic replacement for primary tumours around the knee: experience from Peking University. J Bone Joint Surg (Br).

[20] Shehadeh A, Noveau J, Malawer M, Henshaw R (2010). Late complications and survival of endoprosthetic reconstruction after resection of bone tumors. Clin Orthop Relat Res.

[21] Flint MN, Griffin AM, Bell RS, Ferguson PC, Wunder JS (2006). Aseptic loosening is uncommon with uncemented proximal tibia tumor prostheses. Clin Orthop Relat Res.

[22] Gosheger G, Gebert C, Ahrens H, Streitbuerger A, Winkelmann W, Hardes J (2006). Endoprosthetic reconstruction in 250 patients with sarcoma. Clin Orthop Relat Res.

[23] Healey JH, Morris CD, Athanasian EA, Boland PJ (2013). Compress knee arthroplasty has 80% 10-year survivorship and novel forms of bone failure. Clin Orthop Relat Res.

[24] Pala E, Henderson ER, Calabro T, Angelini A, Abati CN, Trovarelli G, Ruggieri P (2013). Survival of current production tumor endoprostheses: complications, functional results, and a comparative statistical analysis. J Surg Oncol.

[25] Bus MP, van de Sande MA, Fiocco M, Schaap GR, Bramer JA, Dijkstra PD. What Are the Longterm Results of MUTARS® Modular Endoprostheses for Reconstruction of Tumor Resection of the Distal Femur and Proximal Tibia? Clin Orthop Relat Res. 2015. doi:10.1007/s11999-015-4644-8. 10.1007/s11999-015-4644-8 PMC5289150 26649558

[26] Back DL, David L, Hilton A, Blunn G, Briggs TW, Cannon SR (2008). The SMILES prosthesis in salvage revision knee surgery. Knee.

[27] Barrack RL, Lyons TR, Ingraham RQ, Johnson JC (2000). The use of a modular rotating hinge component in salvage revision total knee arthroplasty. J Arthroplasty.

[28] Barrack RL (2001). Evolution of the rotating hinge for complex total knee arthroplasty. Clin Orthop Relat Res..

[29] Berend KR, Lombardi AV (2009). Distal femoral replacement in nontumor cases with severe bone loss and instability. Clin Orthop Relat Res.

[30] Jones RE, Barrack RL, Skedros J (2001). Modular, mobile-bearing hinge total knee arthroplasty. Clin Orthop Relat Res..

[31] Jones RE, Skedros JG, Chan AJ, Beauchamp DH, Harkins PC (2001). Total knee arthroplasty using the S-ROM mobile-bearing hinge prosthesis. J Arthroplasty.

[32] Lombardi AV, Mallory TH, Eberle RW, Adams JB (1997). Rotating hinge prosthesis in revision total knee arthroplasty: indications and results. Surg Technol Int..

[33] Petrou G, Petrou H, Tilkeridis C, Stavrakis T, Kapetsis T, Kremmidas N, Gavras M (2004). Medium-term results with a primary cemented rotating-hinge total knee replacement. A 7- to 15-year follow-up. J Bone Joint Surg Br.

[34] Pour AE, Parvizi J, Slenker N, Purtill JJ, Sharkey PF (2007). Rotating hinged total knee replacement: use with caution. J Bone Joint Surg Am.

[35] Pradhan NR, Bale L, Kay P, Porter ML (2004). Salvage revision total knee replacement using the Endo-Model rotating hinge prosthesis. Knee.

[36] Rand JA, Chao EY, Stauffer RN (1987). Kinematic rotating-hinge total knee arthroplasty. J Bone Joint Surg Am.

[37] Springer BD, Hanssen AD, Sim FH, Lewallen DG (2001). The kinematic rotating hinge prosthesis for complex knee arthroplasty. Clin Orthop Relat Res..

[38] Springer BD, Sim FH, Hanssen AD, Lewallen DG (2004). The modular segmental kinematic rotating hinge for nonneoplastic limb salvage. Clin Orthop Relat Res..

[39] Utting MR, Newman JH (2004). Customised hinged knee replacements as a salvage procedure for failed total knee arthroplasty. Knee.

[40] Westrich GH, Mollano AV, Sculco TP, Buly RL, Laskin RS, Windsor R (2000). Rotating hinge total knee arthroplasty in severly affected knees. Clin Orthop Relat Res..

